# Schroth physiotherapeutic scoliosis-specific exercises for adolescent idiopathic scoliosis: how many patients require treatment to prevent one deterioration? – results from a randomized controlled trial - “SOSORT 2017 Award Winner”

**DOI:** 10.1186/s13013-017-0137-8

**Published:** 2017-11-14

**Authors:** Sanja Schreiber, Eric C Parent, Doug L Hill, Douglas M Hedden, Marc J Moreau, Sarah C Southon

**Affiliations:** 1 Faculty of Rehabilitation Medicine, University of Alberta, Edmonton, Alberta Canada; 2 Department of Physical Therapy, University of Alberta, Edmonton, Alberta Canada; 3 Department of Surgery, University of Alberta, Edmonton, Alberta Canada

**Keywords:** Physiotherapeutic scoliosis specific exercises, Number needed to treat, Clinical significance, Schroth, Exercise, Radiography, Cobb angle, Scoliosis, Adolescents, Spinal curvatures

## Abstract

**Background:**

Recent randomized controlled trials (RCTs) support using physiotherapeutic scoliosis-specific exercises (PSSE) for adolescents with idiopathic scoliosis (AIS). All RCTs reported statistically significant results favouring PSSE but none reported on clinical significance. The number needed to treat (NNT) helps determine if RCT results are clinically meaningful. The NNT is the number of patients that need to be treated to prevent one bad outcome in a given period. A low NNT suggests that a therapy has positive outcomes in most patients offered the therapy. The objective was to determine how many patients require Schroth PSSE added to standard care (observation or brace treatment) to prevent one progression (NNT) of the Largest Curve (LC) or Sum of Curves (SOC) beyond 5° and 10°, respectively over a 6-month interval.

**Methods:**

This was a secondary analysis of a RCT. Fifty consecutive participants from a scoliosis clinic were randomized to the Schroth PSSE + standard of care group (*n* = 25) or the standard of care group (*n* = 25).

We included males and females with AIS, age 10–18 years, all curve types, with curves 10°- 45°, with or without brace, and all maturity levels. We excluded patients awaiting surgery, having had surgery, having completed brace treatment and with other scoliosis diagnoses. The local ethics review board approved the study (Pro00011552).

The Schroth intervention consisted of weekly 1-h supervised Schroth PSSE sessions and a daily home program delivered over six months in addition to the standard of care. A prescription algorithm was used to determine which exercises patients were to perform. Controls received only standard of care.

Cobb angles were measured using a semi-automatic system from posterior-anterior standing radiographs at baseline and 6 months.

We calculated absolute risk reduction (ARR) and relative risk reduction (RRR). The NTT was calculated as: NNT = 1/ARR. Patients with missing values (PSSE group; *n* = 2 and controls; *n* = 4) were assumed to have had curve progression (worst case scenario). The RRR is calculated as RRR = ARR/CER

**Results:**

For LC, NNT = 3.6 (95% CI 2.0–28.2), and for SOC, NNT = 3.1 (95% CI 1.9–14.2). The corresponding ARR was 28% for LC and 32% for the SOC. The RRR was 70% for LC and 73% for the SOC. Patients with complete follow-up attended 85% of prescribed visits and completed 82.5% of the home program. Assuming zero compliance after dropout, 76% of visits were attended and 73% of the prescribed home exercises were completed.

**Conclusions:**

The short term of Schroth PSSE intervention added to standard care provided a large benefit as compared to standard care alone. Four (LC and SOC) patients require treatment for the additional benefit of a 6-month long Schroth intervention to be observed beyond the standard of care in at least one patient.

**Trial registration:**

NCT01610908 April 2, 2012

## Background

The evidence on physiotherapeutic scoliosis specific exercises (PSSE) is rapidly growing and getting stronger. Several randomized controlled trials (RCT) investigating the effect of PSSE in adolescents with idiopathic scoliosis (AIS) have been published. Monticone et al., in their long-term RCT found that PSSE consisting of active self-correction and task-oriented exercises, consistent with Scientific Exercise Approach to Scoliosis (SEAS) [1, 2] improved Cobb angles by 5.3° at skeletal maturity in patients with AIS, while traditional exercises were associated with stable curves. [3] Kuru et al., in their 6-month long RCT [4] in patients with AIS concluded that Schroth PSSE improved the Cobb angle by 2.5° in the supervised group, while the patients who were not supervised deteriorated by 3.3° which was similar to controls (deteriorate by 3.1°) receiving no treatment [4]. Our RCT found that patients with AIS receiving Schroth PSSE for six months in conjunction with standard of care consisting of observation or bracing improved curves by 1.2°, while the controls receiving only standard of care deteriorated by 2.3°. [5] Monticone et al. in an RCT conducted on adults with idiopathic scoliosis with childhood onset undergoing a 20-week long SEAS PSSE or usual physiotherapy, found that the Cobb angle in the experimental group improved by 2.9° and in the control group deteriorated by 2.1° [6]

All these RCTs reported statistically significant results favouring PSSE but none reported on clinical significance. Assessing the clinical significance is important to interpret the results in a way, which is understandable to the end users (clinicians and patients), as well as to facilitate appropriate knowledge transfer. [7–10]

Reporting statistical test results should be supplemented with methods for determining clinically significant change [11–14]. The concept of “clinical significance” basically consists of determining whether “this matters in the real world of clinical medicine.” [9] Several definitions have been proposed for “clinically meaningful effect”. One definition is “the extent to which therapy moves someone outside the range of scores typical of the dysfunctional population or within the range of the functional population” [12]. Another one states that “a clinically significant change is a difference score that is large enough to have an implication for the patient's treatment or care” [15]. These definitions are not associated with a definite statistic to determine the meaningfulness of an observed improvement or deterioration over time with clinical treatment. Therefore, in an effort to evaluate whether the treatment has had a clinically significant effect on a patient’s outcome variable, several approaches have been proposed.

Researchers most commonly assess the minimal clinically important difference (MCID), and use it as a threshold for determining if a patient experienced meaningful change. The MCID is “the smallest difference in score in the domain of interest that patients perceive as important, either beneficial or harmful, and which would lead the clinician to consider a change in the patient’s management” [7]. Two methods are used to establish the MCID: anchor- and distribution-based. Anchor-based methods use an external indicator (anchor) to assign participants into several groupings reflecting the importance/magnitude of their changes in outcome measurement (target). [16] The most commonly used anchor is the Global Rating of Change (GRC) [17]. Distribution-based methods rely on statistical properties of the outcome tool of interest to quantify how much change is deemed clinically important.

There is still no consensus as to which method is the most appropriate. [15, 18] Anchor-based methods because of their reliance on the GRC can be affected by recall bias. [18] Patients often do not remember accurately their health status before the treatment. Distribution-based methods depend only on the reliability of the measurement tool and generally ignore whether outcome changes are perceived as important. [18]

The number needed to treat (NNT) is the number of patients that need to be treated to prevent one bad outcome in a period. A low NNT indicates that a therapy has a positive outcome in most patients offered the therapy. The NNT is easier to interpret by the public than probabilities, and is therefore a useful measurement of the clinical effect.

Therefore, the objective of this secondary analysis of data from a RCT [5, 19, 20]study was to determine how many patients require Schroth PSSE added to standard care (observation or brace treatment) to prevent one progression of the Largest Curve (LC) by more than 5° or of the Sum of Curves (SOC) by more than 10° over a 6-month interval.

## Methods

### Design

This was a secondary analysis of a parallel group RCT on the effect of 6-month long Schroth PSSE intervention added to standard of care compared to standard of care alone (observation or bracing) on the change in Cobb angle in patients with AIS.

### Setting and participants

Fifty consecutive participants with AIS were recruited from a local scoliosis clinic by an independent research coordinator. We included male and female adolescents with AIS, age 10–18 years, all curve types, with curve magnitudes 10° - 45°Cobb angle, with or without brace, and all maturity levels (Risser = 0–5). Surgical candidates, adolescents who had had surgery, had completed a brace treatment and patients with diagnosis other than AIS were excluded. The local ethics review board approved the study, participants provided assent and parents’ provided consent.

### Randomization

Participants were randomized using a computer-generated sequence in pre-sealed envelopes into the experimental or the control group, so that each group included 25 participants. Random block sizes varying between 4 and 8 and stratification for curve types were used. The sequence was prepared by an independent research assistant.

### Intervention

The Schroth intervention consisted of weekly 1-h long supervised Schroth exercises sessions combined with a 30–45-min long daily home exercise program delivered over six months in addition to the standard of care. An exercise prescription algorithm was used to determine which exercises patients were to perform [21]. Schroth PSSE, consist of passive and active postural auto-correction exercises done repeatedly. Exercises are progressed from lying, sitting, or standing positions and from most to least passive support per a review of the quality of the performance demonstrated during supervised visits. Controls received only standard of care (observation or bracing) during the trial time. A detailed description of the intervention has been published previously. [5, 19, 20]

### Outcomes and follow-up

Radiographic measurements included the largest curve (LC) and sum of the curves (SOC) measured by the Cobb angle. The *Cobb angle* is the angle between the upper endplate of the most tilted upper end vertebrae and lower endplate of the lower end vertebrae of the scoliosis curvature observed on a posterior-anterior radiograph [22]. Our semi-automated digital measurement demonstrated excellent reliability with a standard error of measurement (SEM) of ≤2.5°, which is better than most published values for Cobb angle measurements. [23] The intra- class correlation coefficient for estimating the intra-rater reliability was ICC = 0.99 (CI 0.987–0.992). One evaluator extracted all Cobb angles larger than 10° while blinded to groupings, image time points, prior measurements and subject identity. The largest Cobb angle measured was used as the LC and the sum of all Cobb angles exceeding 10° was used as the SOC outcome in the analyses.

### Statistical analysis

Numbers needed to treat (NTT) were calculated as: $NNT=\frac{1}{CER- EER}$, where CER is the control event rate and EER the experimental event rate. The CER and EER are the proportion of patients in the control and experimental group, respectively, who deteriorated by >5° for the LC or by >10° for the SOC. The Wilson score method was used for calculating the 95% CI for the NNT. This was justified because the most frequently used Wald method has several documented shortcomings, including dependency on sample size and producing unreliable and theoretically impossible results when event probabilities are close to 0 and 1 [24, 25]. The Wilson score method provides improved CI and interpretation compared to the Wald method. Tandberg’s calculator for confidence intervals for the NNT was used [26].

We also provided absolute risk reduction (ARR) and relative risk reduction (RRR). ARR is calculated as the difference between the CER and the EER and describes how much better or worse one treatment is at preventing a curve progression by >5° (LC) or 10° (SOC). The RRR, calculated as the ARR divided by the CER, represents how much the treatment reduced the risk of curve deterioration by >5° (LC) or 10° (SOC) relative to the observations in the control group. [27] Intent to treat analysis was used. Patients with missing values were assumed to have had curve progression (worst case scenario).

#### Sample size calculation

This RCT was powered to detect a 0.50 effect size when comparing the change in the primary outcome between two groups with 80% power using a two-tailed 0.05 hypothesis test, and considering a 0.6 correlation between repeated measures in two time points, 50 patients per group were needed. [28] However, the study ended after recruiting 50 participants when funding was received to continue the study as a multicenter RCT with slightly different participants’ criteria (Trial registration NCT01610908). [5]

## Results

The 25 participants in each group were recruited between April 2011 and November 2013. Patients in both groups had similar baseline characteristics and calculated risk of progression by Lonstein and Carlson formula [29] (Table 1). Two participants from the Schroth PSSE group and four from the control group dropped out, due to time constraint (PSSE group), relocation (one control) and travelling for a long time during the trial (one control). Patients with complete follow-up attended 85% of prescribed visits and completed 82.5% of the home program. Assuming zero compliance after dropout, 76% of visits were attended and 73% of the prescribed home exercises were completed. No adverse events were observed in this RCT.

**Table 1:** Baseline characteristics of the study participants

	Schroth exercises + Standard of care (95% Confidence interval), *n* = 25	Standard of care (95% Confidence interval), *n* = 25
Age (years)	13.5 (12.7–14.2)	13.3 (12.7–13.9)
Girls n (%)	23 (92)	24 (96)
Braced participants n (%)	17 (68)	17 (68)
Height (m)	1.60 (1.6–1.6)	1.60 (1.6–1.6)
Weight (kg)	45.9 (42.6–49.1)	50.5 (47.1–54.0)
Largest curve (°)	29.1 (25.4–32.8)	27.9 (24.3–31.5)
Sum of curves (°)	48.1 (39.1–57.2)	54.3 (44.9–63.6)
Risser sign (0 to 5)	1.76 (1.10 to 2.45)	1.44 (0.77 to 2.11)
Lonstein and Carlson Risk of progression [29] (%)	65	65

Baseline and 6-month mean scores for LC and SOC are presented in Table 2.

**Table 2:** Mean scores for each outcome at baseline and 6-month follow-up

Outcome	Group	Mean	Standard Deviation	95% Confidence Interval	Minimum	Maximum
Largest Cobb at Baseline (^o^)	Control*	27.9	8.8	24.3–31.5	11.7	42.0
	Experimental*	29.1	8.9	25.4–32.8	11.3	44.3
	Total	28.5	8.8	26.0–31.0	11.3	44.3
Sum of Curves at Baseline (^o^)	Control	54.3	22.6	44.9–63.6	11.7	95.1
	Experimental	48.2	21.9	39.1–57.2	11.3	86.0
	Total	51.2	22.3	44.9–57.5	11.3	95.1
Largest Cobb at 6-months	Control	29.1	8.8	25.0–33.3	12.1	44.7
	Experimental	27.7	8.9	23.8–31.5	14.4	43.9
	Total	28.4	8.8	25.7–31.0	12.1	44.7
Sum of Curves at 6-months	Control	57.5	24.9	45.8–69.1	15.8	102.4
	Experimental	45.7	21.4	36.4–54.9	14.4	80.6
	Total	51.2	23.6	43.9–58.4	14.4	102.4

*Control = Standard of care group; Experimental = “Schroth + standard of care group

There were 4 (16%) patients in the experimental and 1 (4%) in the control group who improved their LC by >5°. The LC remained within 5° for 18 (72%) participants in the experimental and 14 (56%) in the control group; and there were 3 (12%) participants in the experimental and 10 (40%) in the control group whose LC deteriorated by >5° (Table 3). For the LC, the NNT was 3.6 with a 95% CI 2.0 to 28.2.

**Table 3:** Number of participants improved, stable and deteriorated at the 6-month follow-up

	Improved (Cobb angle reduced by ≥5°) number (%)	Stable (Cobb angle within 5°) number (%)	Deteriorated (Cobb angle increased by ≥5°) number (%)
Largest Cobb			
Schroth + standard of care	4 (16)	18 (72)	3 (12)
Standard of Care	1 (4)	14 (56)	10 (40)
	Improved (Cobb angle reduced by ≥10°) number (%)	Stable (Cobb angle within 10°) number (%)	Deteriorated (Cobb angle increased by ≥10°) number (%)
Sum of Curves			
Schroth + standard of care	1 (4)	21 (84)	3 (12)
Standard of care	1 (4)	13 (52)	11 (44)

One (4%) participant in the experimental and 1 (4%) in the control group improved their SOC by >10°. The SOC remained within 10° for 21 (84%) participants in the experimental and 13 (52%) in the control group; and there were 3 (12%) participants in the experimental and 11 (44%) in the control group for whom the SOC deteriorated by >10° (Table 3). For the SOC, the NNT was 3.1 with a 95% CI 1.9 to 14.2. (Table 4).

**Table 4:** Numbers needed to treat (NNT) with 95% confidence intervals (CI), Absolute risk reduction (ARR) and Relative risk reduction (RRR)

	NNT (n, 95% CI)	Absolute Risk Reduction (%)	Relative Risk Reduction (%)
Largest Cobb Angle	3.6 (2.0 to 28.2)	28	70
Sum of curves	3.1 (1.9 to 14.2)	32	73

The ARR indicated that, with the Schroth intervention, there was a 28% and 32% absolute reduction in the risk of curve progression over the respective clinically important thresholds associated with the LC and SOC, respectively (Table 4). Further, the RRR indicated that the Schroth intervention reduced the risk of curve deterioration in excess of 5° for the LC by 70% and exceeding 10° for the SOC by 73% (Table 4).

## Discussion

This first study to assess the NNT in patients with AIS undergoing Schroth exercise intervention demonstrated the clinically important effect of the Schroth intervention. We calculated that over a 6-months period one additional person will avoid LC deterioration by >5° or SOC deterioration by >10°, respectively, for every four participants undergoing Schroth PSSE intervention in addition to standard of care compared to receiving standard of care only.

While the health consequences of progression of LC by >5° or of the SOC by >10° over six months are not fully documented, it is clear that the goal of every scoliosis therapy is to stop the curves from progression into a range where a change in care would be warranted. For a patient under observation, progression of the LC by >5^o^ might lead to a brace prescription, and for a participant in brace, progression might warrant a modification of the brace prescription or a recommendation for surgery. Therefore, avoiding a more aggressive treatment even if only for six months is clinically meaningful.

Monticone et al. [2] also reported results favouring SEAS PSSE at maturity. None of the patients from the SEAS PSSE group deteriorated by >5^o^, 62% improved, and 38% remained stable suggesting that 100% were treated successfully [2]. In the control group receiving general physiotherapy, none improved, 4 (8%) deteriorated, and 47 (92%) remained stable. We calculated NNT based on those results as 12.8 with CI containing “0”, suggesting that the treatment effect was not significant. However, there were 61.5% more patients who improved beyond 5° in the PSSE group, clearly suggesting the benefit of PSSE over the long term. This lack of clinical importance in the treatment effect in this study is not clinically significant in terms of prevention of deterioration by >5°, could be contributed to the fact that the girls under investigation had low risk of progression (35% risk of progression according to Lonstein and Carlson’s formula) [30], and the comparator was an active therapy.

While ours was the first study reporting NNT for exercise treatment, NNTs have been reported for brace management. The Bracing in Adolescent Idiopathic Scoliosis Trial (BrAIST) study, which was partly a randomized and partly a patient-preference trial, reported that 3.0 (95% CI, 2.0 to 6.2) patients needed to be treated in order to prevent one case of curve progression warranting surgery based on the results from the randomized cohort and applying intention-to-treat methodology. [31] This is not in line with Sanders et al. [32] results in a prospective follow-up of a cohort of 126 immature patients (Risser 0–2) with AIS and curves between 25° and 45° treated with a Boston brace and brace wear to the end point of progression to surgery. The noncompliant patients were compared both with highly compliant patients and with the entire cohort. The authors reported a NNT of 7 to avoid surgery. However, the 95% CI included “0”, indicating that the treatment effect was not significant, so the NNT cannot be directly interpreted. For the highly compliant patients (bracewear >14 h) compared with non-compliant patients, the NNT (3.0; 95% CI, 2.0 to 7.0) was similar to that reported in the BrAISt and the present study.

Nachemson and Peterson multicenter study compared observation and bracing for girls with thoracic major curves and Cobb angles of 25° to 35°. [33] In their study they found that 17 of 88 braced patients with full follow-up deteriorated by >6°, and 58 of 120 observed patients, which gives NNT of 3.4 (95% CI 2.5 to 6.2). However, in the worst-case analysis when the dropouts are considered failures, there were 36% failures among braced patients and 56% among observed cohort, producing the NNT of 6.3 (3.6 to 30.3).

Danielsson et al. used some of the subjects from Nachemson and Peterson study, and determined the treatment failure at a mean of 16-year follow-up, found that none of the 41 initially braced patients progressed by >6°, whereas 40% of those initially under observation progressed. However, there was no difference in progression between the two groups after maturity (5.7° in the braced group and 7.0° in the observed patients). The authors concluded that since “70% of the observed patients during the original study period did not require any other treatment, 70% of the initially braced patients can therefore be regarded as having been treated unnecessarily.” [34] Of the remaining observed patients, who needed a treatment, only 10% required surgery, meaning that 10 patients need to be brace-treated to avoid 1 surgery (NNT = 10; 95% CI 5.7 to 23.9).

In our sample, 17 (68%) patients wore a brace in each group. When a good compliance was assumed as brace wear of >16 h, there were 8 (47%) compliant patients in the Schroth PSSE group and 7 (41%) in the control group. There were 20 (80%) patients in the experimental group with >75% compliance with daily home exercise program. Of those who wore a brace in the Schroth PSSE group, 8 (47%) non-compliant and 7 (41%) compliant patients with brace had improved or stable curves. There was 1 (6%) compliant and 1 (6%) non-compliant patient with a brace who deteriorated. Of the 5 (20%) patients with <75% compliance with the home exercise program, 3 (60%) did not have a brace; of 2 that did have a brace, 1 (50%) was compliant with it and 1 (50%) was not. Of the eight patients in the Schroth PSSE group who did not wear a brace 7 (88%) improved, and 1 (12%) deteriorated.

While our sample size was relatively small, the distribution of compliant and non-compliant patients with a brace between the groups was similar. In addition, there was a similar number of compliant and non-compliant patients with brace who improved or deteriorated in the experimental group. Therefore, the compliance with a brace did not have a direct influence on the effect of the intervention, as long as the patients were compliant with the exercises. However, this study did not compare Schroth PSSE with bracing alone, so this should be interpreted with caution.

There is a general consensus that current brace indications lead to overtreatment. [32, 34] Many patients wearing a brace do not need to wear one because their curves would naturally not increase to the surgical range, and only estimated 10% of braced patients would avoid surgery. [34] We showed that four participants undergoing Schroth PSSE intervention in addition to standard of care and not standard of care only would need to be treated in order to see one additional curve improvement over a 6-months period.

We observed that adding Schroth PSSE might address a need and offer a treatment complement in patients who are not fully compliant with brace treatment. In our sample, despite promoting both exercise and brace compliance, of nine patients reporting wearing their a brace less than 16 h a day in the exercise group, 8 (89%) were highly compliant with exercises.

Our study was designed to determine whether adding Schroth PSSE to standard of care (observation and bracing) would lead to better outcomes, as compared to standard of care alone. To assess the differences between brace vs. exercises alone, we would need to deny the brace treatment to the patients who meet the SRS bracing criteria, which would raise ethical concerns. Despite the clinically significant results in our study, the overarching question of who will benefit from wearing a brace, who from doing the Schroth PSSE, and who from a combined treatment still remains.

### Limitations

A limitation of this study is its short-term follow-up. Therefore, we cannot draw conclusions regarding the effects of a longer period of treatment, and cannot answer the question “how many patients need to be treated with Schroth PSSE added to standard of care to prevent one surgery or prevent the need for a brace?” However, our study shows that the Schroth PSSE intervention added to standard care consisting of bracing or observation can delay the time where a more aggressive scoliosis management is indicated.

The small sample size precluded us from conducting subgroup analysis related to compliance, curve type, baseline severity or maturity. Interestingly, of 25 patients in the exercise group, 20 reported >75% compliance with home exercise program. In the control group, there were 17 patients who wore braces. Of those, only seven were considered compliant as they wore their braces >16 h/day. This might have resulted in a larger number of deteriorated patients in the control group as compared to the exercise group.

Small sample size also affected confidence intervals of the NNTs. Regardless, of the wide CI, the treatment was clinically important, because the CI were not disjointed (did not contain “0”).

While the patients and therapists could not be blinded to treatment, our outcomes’ assessors were blinded.

Participation in the Schroth intervention may have had an effect on brace wear compliance likely illustrating the importance of team-work.

## Conclusions

The short-term Schroth PSSE intervention added to standard care provided a clinically important benefit illustrated by low NNT when compared to standard care alone. Results suggest that four (LC, SOC) patients require treatment for the additional benefit of a 6-month long Schroth PSSE intervention added to standard of care to be observed beyond the standard of care in at least one patient.

## Abbreviations

AIS: Adolescent Idiopathic Scoliosis
ARR: Absolute Risk Reduction
BrAIST: Bracing Adolescent Idiopathic Scoliosis Trial
CER: Control Event Rate
CI: Confidence Interval
EER: Experimental Event Rate
ICC: Intra-class Correlation Coefficient
LC: Largest Curve
MCID: Minimal Clinical Important Difference
n: Number
NNT: Numbers Needed to Treat
PSSE: Physiotherapeutic Scoliosis-Specific Exercises
RCT: Randomized Controlled Trial
RRR: Relative Risk Ratio
SEAS: Scientific Exercise Approach for Scoliosis
SEM: Standard Error of Measurement
SOC: Sum of Curves
